# Intercalating the Role of MicroRNAs in Cancer: As Enemy or Protector

**DOI:** 10.31557/APJCP.2020.21.3.593

**Published:** 2020-03

**Authors:** Tapan Behl, Chanchal Kumar, Rashita Makkar, Amit Gupta, Monika Sachdeva

**Affiliations:** 1 *Chitkara College of Pharmacy, Chitkara University, Punjab, *; 2 *Vallabhbhai Patel Chest Institute, University of Delhi, Delhi, India, *; 3 *Fatima College of Health Science, AI Ain, UAE. *

**Keywords:** Carcinogenic, tumor suppressor genes, metastasis, leukemia, apoptosis

## Abstract

**Objective:**

The transformation in cells at genetic levels stimulatesthe proliferation of cancer. The current review highlights the role of miRNA in management of cancer by altering processes of body at cellular levels.

**Methods:**

A deep research on the literature available till date for miRNA in cancer was conducted using various medical sites like PubMed, MEDLINE from internet and data was collected. The articles were majorly preferred in English language.

**Results:**

The development of normal cells into cancerous cells is a multivalent procedure highlighting numerous responsible factors. During the progression of cancer, the role of oncogene and tumor suppressor genes outshines at different levels of tumorogenesis. Metastasis poses highest threat in cancer progression and fabricates obstacles to clinicians and researchers in preventing formation of tumor on secondary sites. The mesenchymal-epithelial transition (MET) and epithelial mesenchymal transition (EMT) induce dissemination and ultimately progression of cancer.

**Conclusion:**

A comprehensive knowledge of the altered genes and the mechanism by which they induce formation of tumor is essential as they contribute in proliferating cancer at various stages, aggravating clinical symptoms. Hence miRNAs can be efficiently employed as an emerging treatment therapy for cancer.

## Introduction

Cancer in itself is a very broad term and condition. It can be described as a disease in which the cellular modifications in the body lead to uncontrolled abnormal growth and proliferation of the cells that accumulate and protrude towards tumor formation. There is not always rapid cell growth but cell growth may be suppressed in certain scenarios as well. The tumor growth cancers can be detected much easily when compared to malignant like blood caners which are hard to diagnose. In a study in USA in 2014, it has been estimated that over 480,000 people die with cancer each year. It is still one of the most debilitating disease worldwide and therapy advances in management of cure of cancer are much needed.

Alterations in genes with simultaneous modification in phenotype of the individual are linked with metastasis of cancer cells (Yokota, 2000). The non-coding miniature endogenous RNAs also called micro RNAs (miRNAs) regulates a wide array of metabolic processes that are indulged in induction of carcinogenesis. MiRNAs were primarily revealed in *C. elegans*, and were later established for their presence in human and other eukaryotes (Lee and Ambros, 1993; Perron and Provost, 2008). It has also been assumed that the human genome comprises of nearly 1-5% of miRNAs which evidently regulates about 30% of genes that code for specific proteins (Berezikov et al., 2005; Rajewsky, 2006). Various studies established the significant functioning of miRNA in regulating genetic expression of metabolic and cellular origin pathways (Esau et al., 2006; Krutzfeldt et al., 2005). de Croce’s team was the first to state the part of miRNA in proliferating cancer and identified genes that suppress tumor in chronic stage of B-cell lymphocytic leukemia. In latter cases, miR-16-1 and miR-15a, two major miRNAs downregulated (Calin et al., 2002). Further studies conducted stated that they not only suppressed the growth of tumor but also promoted apoptosis via manipulation of Bcl-2 and anti-apoptotic proteins, hence increasing expression of non dividing malignant B cells (Cimmino et al., 2005). The miRNA manifestation is highly regulated by transcription factors. Hence, miRNA illustrationscould bealtered by modifying the factors responsible for transcriptionsuch as p53 and c-Myc. In malignant cases c-Myc is elevated and guides cell proliferation by stimulating transcription (O’donnell et al., 2005). 

*Biogenesis of miRNA*

MicroRNAs (miRNAs) comprise of a long 20–22 nucleotide chain of non-translating RNAs. About 1–5% of miRNA genome undergoes transcription on interaction with enzyme RNA-polymeraseII (Lee et al., 2002; Cai et al., 2004). Biogenesis is a long procedure and occurs at nucleus and cytoplasmic compartments. The transcribed genes initiate capped 5’ end and polyadenylated 3’ end generating the first primary transcript namely pri- miRNA. Its a large stem-loop like formation and fabricates the formation of single stranded 5’ and 3’ RNA that projects theformation of functional miRNA (Zeng and Cullen, 2005). Pri-miRNA further branches to precursor miRNA (Pre-miRNA) via nuclear RNAse III or Drosha comprising complex microprocessors along with DGCR8. The pre-miRNA formed migrates to cytoplasm via GTP complex Exportin-5 and Ran from nucleus (Bohnsack et al., 2004). The latter on reaction with dicer associated TRBP or TAR RNA-binding protein and enzyme RNase III generate long double stranded ~22 nucleotide miRNA (O’Donnell et al., 2005). An enzyme named helicase unzips the doublyentangled miRNA and develops two single strands, one of which engages itself in formation of RNA induced silencing complex (RISC) comprising of Argonaute-2 (AGO2) protein degrading other. Upon binding to 3’UTR’s, the miRNA induced silencing complex (miRISC) suppresses translation causing degradation of mRNA targets (Hutvagner et al., 2001). Single miRNA directs numerous pathwaysand have a tendency to extinguish multiple transcripts. Disturbed transcription regulation may synthesize and express abnormal and diversified proteins. Hence, miRNA’sare recognized as important determinants of cell fate in response to cellular expression during cancer progression.

*Involvement of miRNAs in cancer*

Various studies conducted in previous decade clearly explained miRNA and its part in regulating malignancies in humans. Abnormal miRNAs manifestations cause gene dysfunction and are responsible for pathological conditions of various diseases. They have ability to regulate the genetic expression via repression of pre-transcription or directing deletion and destruction of target gene by messenger RNA (mRNA) (Fabian andSonenberg, 2012). Some studies identified miRNAs as a standardized element in transcriptional regulation of metastasis (Liz and Esteller, 2016), arresting cell cycle and inducing apoptosis (Tiwari et al., 2012). In this article we will emphasize thecontribution of miRNA in modulating and abolishing proliferation of cancer and cell survival. 

*Restraint of miRNA in Transcriptional control*

Regulating transcription is an essential factor for cell survival. Convoluted interactions between the controls of transcription and post-transcription proteins regulate gene expression leading to manifestation ofgenes explicit to cell-type patterns ascertained by the stages of development and differentiation of etiological conditions. Biological system directly or indirectly has connected signaling pathways. Theoncogenic and tumor suppressive transcription factors become dysfunctional, resulting in cell transformation due tochanges in transcriptional factors, for example,P-TEFb can be stimulated by c-Mycwhich activates the enzyme RNA polymerase II and maintains transcription (Chalhoub and Baker, 2009). c-Mycis a factor oftranscription thatrepresses and activatestarget genes. A tumor gene suppressor called phosphatase and tensin homology (PTEN) is activated by c-Myc on transcriptionwhich further dephosphorylates PI3K and its products named phosphatidylinositol 3,4,5-triphosphate (PIP3) by inhibiting PI3 kinase mediated growth signals. Failure of expression of PTEN inhibits AKT activation further prohibitingpromotion of proliferation of cells and apoptosis (Rahl et al., 2010). Inhibition of miRlet-7 increases proliferation of cells (Bethel et al., 2009), whereasmanifestation of mature miRlet-7 arrests the cell cycle (Legesse-Miller et al., 2009). Interestingly another miRNA named miR-145is capable to arrest development of tumor cells by aiming c-Myc, mucin-1 and other related genes (Johnson et al., 2007). Contrivedthe miR-143 functioning whilst interacting with native miR-145inhibits the major signaling molecules like p68/p72/β-catenin (Wnt), ERK5/c-Myc, hence preventing the development of intestinal tumor (Sachdeva and Mo, 2010). Therefore, impaired expression of miRNAs could be involved in alteration of transcription factors that may support or cease the cancer development and progression.

*miRNA in cell cycle*

Appropriate regulation of cell cycle assures genetic adherence while mitotic division. Inaccurate cell cycle and disturbed genetic profiles may protrude incomplete replication and arrest cell cycle thereby causing cell apoptosis. The cell cycle is directed by two main molecular mechanisms: a cascade of protein phosphorylation polished by cyclin dependent kinase (CDK) and cyclin complex besides restriction points such as G1/S/G2/, metaphase (Takaoka et al., 2012). The principal hallmark of proliferation and progression of cancer are the mutations in genes due to dysregulated gene expressions andirregular cell cycle. Oncogenic miRNAs wreck CDK inhibitors and assist the succession of entry of cell cycle and suppress transcription of the retinoblastoma family (Chen et al.,2010).

A miR-137 self-manifestation is repressed by hypermethylation and is associated with the expression of CDK6 in oral carcinogenesis cases (Bueno and Malumbres, 2011). The miR-449a/breinforces negative feedback on signaling of Rb/E2F, and stimulate transcription of miR-499a/b by E2F, declining the levels of CDK6 and Cdc25A implied in regulation of cell cycle(Kozaki et al., 2008). A huge emphasis is given on miR-126 as it is beautifully of brews signaling of PI3K via IRS-1 (Yang et al., 2009) VEGF (Zhang et al., 2014). In hypoxic conditions, miR-210 interacts with hypoxia-inducible factor 1-alpha or HIF-1a. HIF-1a mediated cell-cycle arrest antagonizes transcriptional activity of c-Myc transcriptional preventing signaling of canonical Wnt, thereby reducing malignancy (Chen et al., 2009; Huang et al., 2009).

*miRNA in EMT/MET*

The fundamentalcharacter of MET and EMT in invading cancer has recently been investigated. Through various studies it has been found that modification in individuality of cells upon transformationmanifest cancer. Epithelium, a cellular phenotype, acts as a surface barrier and cells possessing invasive and migratory properties are referred as mesenchyme. The incidence of transition of epithelial mesenchymal cells is popular and cells tend to gain migratory properties by exploiting the process besides change in itsadhesive property. Impressive rearrangement of the cytoskeleton of actin besides the simultaneous formation of prominence memberis must for invading proliferation. The cells divide themselves from region of primary tumor and spread around the associated tissues.The cancerous cells through extracellular matrix pursue a chemo alluring pathway as they lose cell to cell contact thereby remodeling adhesion sites of cell-matrix. EMTexhibitsa remarkable attribute in proliferation of cancer and its invasion. Regulating genes by EMTcan prove to be amazing therapeutic targets for management of cancer. Various stages of metastasis express the processes commonly undergone by EMT (Hermeking, 2012). On the other hand, MET is reciprocal of EMT and is fundamental for cellular proliferation. The cells of cancer invade through EMT processes and relapse to epithelial state by MET to proliferate to secondary surrounding sites, hence act smartly (Lo et al., 2017). The markers of EMT are highlighted by failure of adhering cells, repressingexpression of E-cadherin, an associated marker for acquisition of mesenchyma like N-cadherin, Vimentin, and Fibronectin, and elevated motility of cells (Yang and Weinberg, 2008). 

Numerous miRNAs are stated for their indulgence in single or multistep event of metastasis since the past decade. Some of the miRNAsare linked with the EMT like family of miR-205 and mir-200,therefore targeting genes namely SIP1 and ZEB1. The ZEB1,also called deltaEF1 and SIP1 also called ZEB2 are suppressors of E-cadherin (Kalluriand Weinberg, 2009). Suppressing miRNAs issufficient to execute thefamily of miR-200 expressionandavoid EMT induced by TGF-beta (Gregory et al., 2008). Many miRNAs acting as inhibitors and inducers of EMT in management of cancer have also been documented, for example, miR-21 (Ding, 2014), miR-7 (Yang et al., 2009), miR-10b(Han et al., 2012), miR-125b (Han et al., 2014), miR-155 (Hong et al., 2016), miR-9 (Johansson et al., 2013), miR-497 (Gwak et al., 2014), and miR-5003-3p. The examples of miRNA linked with MET include miR-34a, miR-34b and miR-34c (Liu et al., 2016).

*Apoptosis regulated miRNA*

DNA damage induced disturbance or alteration in signaling pathways seize the cycle of cell growth, modifying phenomenon of apoptosis. Apoptosis or commonly called programmed cell death is widely studied and targeted phenomenon in cancer research. Itirreversibly kills the damaged cells and exhibits beneficial effects in organism. The apoptotic events occur through equilibrium between the pro- and anti-apoptotic family of proteins (Brighenti, 2015). Apoptosis is induced by changes in physiological or pathological conditions in body or in response tocertain stimuli. The enzyme p53 exhibits a significantrole in apoptosis induced death of cells which wasoriginallystatedin year 1991 by M. Oren with the help of *p53* gene which lacks cell line of murine myeloid leukaemia. Apoptosis can be classified under two categories: “intrinsic” and “extrinsic” (Shortt and Johnstone, 2012). The former path requires Cytochrome Cfor recruiting procaspase-9 complex. The pathway for apoptosis is moderately influenced by bcl family members linked to mitochondrial membrane (Elmore, 2007; Shamas-Din et al., 2013). The Bcl-2-antagonist/killer namely Bak and Bax are involved in retotion to the apoptotic impetus (Czabotar et al., 2014; Luna-Vargas and Chipuk, 2016). The extrinsic pathway sparks on activation of receptors of dependence and death(Flusberg andSorger, 2015; GibertandMehlen, 2015). The ligands for receptors of dependence and death bind to their relative membrane receptors, likeFas ligand (FasL), tumor necrosis factor (TNF-α), TNF-like weak inducer of apoptosis (TWEAK) and TNF-related inducing ligand (TRAIL) (Wajant, 2002). Death receptor ligands stimulate the death inducing signaling complex (DISC), that further activates CASP8 and regulate its function (Verbrugge et al., 2010). The dependence receptor family consist, netrin 1 (NTN1), unc-5netrin receptor A (UNC5A) and the neurotrophin receptor tyrosin kinase 3 (NTRK3). 

Various reports have suggested participation of miRNA in functioning and apoptosis of cell. Thorough studies showhigh manifestations of miR-221 and miR-222 which tends to regulate extrinsic pathways via TRAIL induced apoptosis of cell (Dickens et al., 2012). The miR-K10 is an onco-miR and encourages tumor cell existence by down- regulating TNF-like weak apoptosis inducing (TWEAK) receptors (Miller et al., 2008). Similarly, decreased concentrations of miR-15 and miR-16 assisst Bcl2 expression in the Chronic lymphocytic leukemia (CLL) and activate apoptotic pathway signaling and can be strategizedas an approach for treatment of tumors due to over expression of *Bcl2*. The miR-145 via dependent TP53 manner has been identified in establishing death induced pro-apoptotic effect. miR-145 on stimulation activate TP53 level and increase their levels which inversely proliferate the tumor grades in cancer of breasts (Abend et al., 2010). Restoring *miR-133a* expression suppresses tumorigenicity in bone cell sarcoma via cell apoptosis (Schanenand and Li, 2010). Augmented concentrations of miR-210 in hypoxic conditions induce existence of cells (Ji et al., 2013; Thiery et al., 2009).

*miRNA in therapeutics*

To fabrictate therapeutic strategies, a thorough knowledge ofvarious mechanisms like transcription regulation, epigenetic or chromosomal changes and miRNAs mediated defects in cancer progression is must. Since past decademiRNAs have been in limelight and attention for their targettingto develop new treatment approaches. The miRNA are fundamentally active in major cellular functions like proliferation, differentiation, growth and metabolism.The miRNAs are also engaged in cancer biology and asa regulatory genetic element. Studies done till date pose miRNA to exhibit mainpart in proliferating tumor malignancy by acting as oncogenes modulator or tumor suppressor element. Different miRNA express and flourish development of individual diseases on alterations. Fate of the disease and functioning of genes is regulatedby overexpression or down expression of miRNA in varied medical complications (Pichiorri et al., 2010). However, restoring their function can reduce or even abolish tumor growth (Hata et al., 2015).

Therefore, miRNA mediated anti-cancer therapy can be usedalone or combined with conventional medicines(Wahid et al., 2010; Lennox andBehlke, 2011). miR-100 possesses chemo-resistance properties incancerous cells of lungs (Price and Chen, 2014). Hindering epigenetics of miR-199b-5p increases the sensitivity to drug in ovarian cancer resistant to chemotherapy (Xiao et al., 2014). miR-21 was established as an onco-miRdisplaying high levelsin cancer, comprising target genes in association like *PDCD4*, *PTEN*, and *Akt/Rb* signaling (SPRY1) (Liu et al., 2014; Meng et al., 2007; Thum et al., 2008). 

Researchers are intended to design adjuvant molecular approach along with chemotherapy to control oncogenic *miRNA* expression and prompt functioning of genes that have ability to suppress tumor (Frankel et al., 2008; Wu et al., 2005; Lee et al., 2004).

In conclusion, alterations in genetic makeup of a human promote cancer cell growth. The carcinoma progression is a multistep process;therefore a deep knowledge the mechanisms at molecular and microenvironmental level that trigger growth of tumorare highly essential.Molecular investigations of cancer cells atvaried levels of cancer progression display the diversity in genes that suppress tumor and other oncogenes correlated with the clinical pugnacity of cancer and its proliferation. A decade of research and pieces of evidence suggest the emergence of micro RNA. Through extensive studies, the precisepathway and functions of the miRNA has been established. Differentially dysregulated miRNA leads to cancer progression and severity. Involvement of miRNA and their expression in cancer related pathwaysmust be understood. However, the role of miRNA role has been defined in regulation of cancer and its related pathways towards malignancy and metastasis of the tumor. The current article explainsmiRNA and itsfunction ina variety of biological events like transcription, metastasis, cycle of cell growth, cancer progression and apoptosis. The emerging scenario suggests that miRNA also have propulsive indication in cell differentiation and act as a critical element in determining fate of cell and fundamental oncogenesis. Therefore, targeting the oncogenic miRNA or revitalization of mi-RNA induced tumor suppression could be a novel concept in advanced therapy. To develop miRNA inhibitors and restoring levels of miRNA, we must determine the principal miRNA that possesses clinical importance at different stages in various cancers.

**Figure 1:**
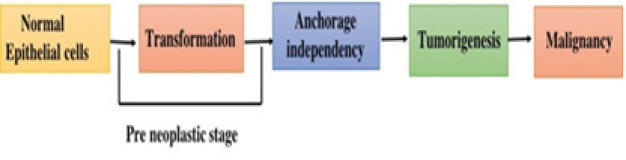
A Multistep Process is Involved in Tumor Formation. Epithelial cells pass the transformation stage on exposure to stimuli and lose anchorage dependency termed as pre neoplastic stage. Anchorage independent cells advance to malignant cancer

**Figure 2:**
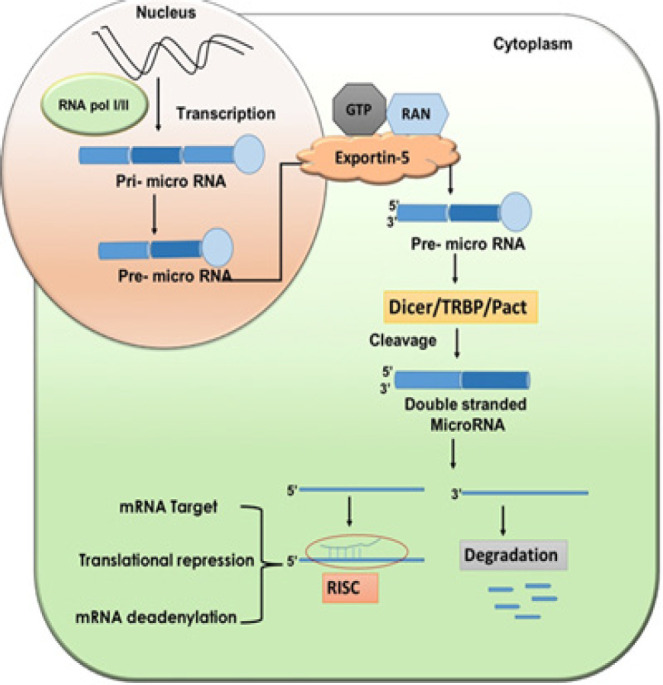
microRNA Biogenesis and Their Maturation: a systematic process of miRNA development and maturation. Only one strand of mature miRNA either 5’ or 3’ will be able to induce formation of RISC which is involved in degradation of target mRNA
